# Metabolomics and metagenomics reveal the impact of γδ T inhibition on gut microbiota and metabolism in periodontitis-promoting OSCC

**DOI:** 10.1128/msystems.00777-23

**Published:** 2024-01-23

**Authors:** Wei Wei, Jing Li, Boyu Tang, Ye Deng, Yan Li, Qianming Chen

**Affiliations:** 1 State Key Laboratory of Oral Diseases, National Clinical Research Center for Oral Diseases, Chinese Academy of Medical Sciences Research Unit of Oral Carcinogenesis and Management, West China Hospital of Stomatology, Sichuan University, Chengdu, Sichuan, China; 2 Department of Prosthodontics, Beijing Stomatological Hospital, School of Stomatology, Capital Medical University, Beijing, China; 3 CAS Key Laboratory of Environmental Biotechnology, Research Center for Eco-Environmental Sciences, Chinese Academy of Sciences, Beijing, China; University of California San Diego, San Diego, California, USA

**Keywords:** γδ T, OSCC, metabolomics, metagenomics, microbiota

## Abstract

During the process of periodontitis-promoting oral squamous cell carcinoma (OSCC), the periodontitis microbiota can facilitate OSCC development by activating γδ T cells. Inhibiting γδ T cells through immunotherapy has been shown to significantly alleviate various types of cancer. However, the underlying mechanism by which inhibiting γδ T cells influenced cancer treatment has not been fully elucidated. In this study, a mouse model of OSCC with periodontitis was established, and γδ T cells were inhibited by antibodies. Gut samples from the mice were collected and analyzed by metabolomics, metagenomics, and 16S rRNA. Integrative analysis of the gut metabolome and microbiome revealed that targeting γδ T resulted in changes in the levels of metabolites associated with cancer in the gut. Although there was no difference in the α diversity of microbiota, β diversity was significantly different, with a more heterogeneous community structure in the mice receiving targeted γδ T immunotherapy. Statistical analysis of the gut microbiota at the species level revealed a significant enrichment of *Lactobacillus murinus*, which was significantly correlated with γδ T abundance. The functional analysis revealed that inhibiting γδ T could impact the functional gene. A comprehensive analysis revealed that *L. murinus* is especially associated with changes in adenine, which also had connection with the concentration of IL-17 and the abundance of γδ T.

**IMPORTANCE:**

This study revealed the effect of γδ T cells on gut microbial dysbiosis and identify potential links between gut microbiota and metabolism, providing new insights into the role of γδ T during the process of periodontitis-induced OSCC, and identifying relevant biomarkers for future research and clinical monitoring protocol development.

## INTRODUCTION

Oral squamous cell carcinoma (OSCC) is a common malignant tumor, with over 90% of cases occurring in squamous tissue of the head and neck. It is characterized by fast metastasis, high recurrence rates, and drug resistance, with over 350,000 cases and ~170,000 deaths from OSCC annually (1, 2). The main risk factors for OSCC include smoking, heavy alcohol consumption, betel nut chewing, and the human papillomavirus (HPV). Additionally, differences in oral microbiota, dietary factors, immune status, environmental pollutants, occupational exposure, and genetic diseases are related to the development of OSCC (3).

Periodontitis is an oral inflammatory disease caused by bacterial plaque microorganisms. In addition to being an oral disease, chronic periodontitis has been shown to develop into a systemic disease (4). Research has shown that there is a correlation between periodontitis and oral cancer (5–7). Periodontitis is one of the risk factors for oral cancer, and patients with periodontitis have a significantly increased risk of developing OSCC (8). Our previous research has also provided strong support for the promotion of OSCC development by periodontitis (9).

γδ T cells are a unique subset of lymphocytes that have been reported to have anti-tumor or pro-tumor functions in several cancer types and play an important role in immune responses (10). As a key subset in oral barrier immune surveillance, γδ T cells can recognize various pathogen antigens (11). IL-17^+^ γδ T cells are the major source of interleukin-17 (IL-17), which has an immunosuppressive effect and can also directly promote cancer progression (12). Our previous research has demonstrated that inhibiting γδT cells has a significant therapeutic effect on periodontitis-promoted OSCC, and γδT cells can be a key target for treating OSCC promoted by periodontitis (9). Research has shown that gut microbiota can inhibit IL-17 production by cecal γδ T cells, and metabolites derived from these microbiota can reduce the production of IL-17 by gut γδ T cells (13). The gut microbiota can shape overall immunity and affect health and disease status, including cancer, at the systemic level (14). The sustained interaction between the gut microbiota and the mucosa regulates inflammation and mediates immune tolerance (15). Changes in the gut microbiota may affect the stability of the oral microbiota composition through the gut-oral axis, and may also affect the occurrence of oral cancer through regulatory effects on the immune system (16).

The mechanism by which inhibiting γδ T cells promotes treatment of OSCC through periodontitis has not been fully elucidated. In this study, we collected and sequenced gut samples from OSCC mice treated with γδ T cell immunotherapy (Fig. S1), hoping to uncover the role of γδ T cells in the gut during the process of periodontitis-promoted OSCC and identify relevant biomarkers for future research and clinical monitoring protocol development.

## MATERIALS AND METHODS

### Collection and processing of oral saliva samples

This experiment also included four healthy volunteers without periodontitis. All volunteers had no other maxillofacial or serious systemic infectious diseases, had not taken antibiotics, hormones, or antifungal drugs within 3 months, had no history of surgery, radiotherapy, chemotherapy, or pregnancy within the past 6 months. At 9 a.m., all participants were asked to rinse their mouths after fasting for 1 hour, and ~2 mL of non-stimulated saliva was collected. After mixing the saliva samples, they were centrifuged at 8,500 g for 10 minutes, and the lower precipitate was aliquoted and quickly frozen for storage. This study was approved and supervised by the Medical Ethics Committee of West China Hospital of Stomatology, Sichuan University (WCHSIRB-OT 2019-015), and all participants gave informed consent.

### Experimental mouse model

Six-week-old male BALB/c mice free of specific pathogens (SPF) were purchased from Chengdu Dossy Experimental Animals Co., Ltd. and were housed in the State Key Laboratory of Oral Diseases at Sichuan University. SPF mice were bred in an environment free of specific pathogens, without common pathogenic infections such as bacteria, viruses, and parasites. The use of SPF mice helps eliminate interference from external pathogens, ensuring more reliable experimental results. The mice were fed with sterile food and water throughout the experiment. All procedures in the animal experiment were performed in accordance with the regulations of the State Key Laboratory of Oral Diseases. This animal study was approved by the Ethics Committee of West China Hospital of Stomatology, Sichuan University (WCHSIRB-D-2019-015).

The AOP-control group (establishment of periodontitis and submucosal injection of squamous cell carcinoma for 21 days, advanced OSCC with periodontitis, *n* = 6) and AOP-treated group (inhibition of γδ T cells by intraperitoneal injection of UC7-13D5 in the AOP-control group, *n* = 5) mice were subjected to periodontitis model treatment: mice were given sterile water containing kanamycin (0.5 mg/mL) for three consecutive days, and then switched to sterile water without antibiotics after oral bacteria washing. A 5-0 sterile suture was tied around the second molar in the maxilla of the mouse. Microbiota inoculation was obtained from saliva of patients with periodontitis or healthy individuals. We collected 2 mL of saliva from each designated patient or healthy person. All saliva was mixed, aliquoted, and cryopreserved quickly. The frozen human saliva sample was centrifuged at 8,500 g for 15 minutes, and the resulting precipitate was thoroughly mixed with 3% carboxymethyl cellulose, ensuring that each mouse is inoculated with an equal volume of the mixture (9). The mixture was repeatedly applied to the cheek and palatal surfaces of the mouse molar area. This was done for seven consecutive times, with 1-day intervals between each inoculation.

The AOP-control and AOP-treated group mice were all subjected to OSCC model treatment: 5 × 10^6^ SCC7 squamous cell carcinoma cells were resuspended in 50 µL DMEM medium and injected into the submucosa of the cheek of the mouse. After inoculation of cancer cells, the number of surviving mice was recorded every day. On the 21st day after cancer cell inoculation, the mice were euthanized, and tumor tissue was collected. The AOP-treated group mice were given intraperitoneal injection of TCR γδ monoclonal antibody (200 mg/mouse) every 2 days until sample collection.

### Liquid chromatography-mass spectrometry metabolomics analysis

Under sterile conditions, fecal samples were collected from each group of mice and stored at −80°C for testing. This test was completed by Guangdong Magigene Biotechnology Co., Ltd. ① After grinding and crushing 25 mg of the sample, 500 µL of extraction solution (40% methanol/40% acetonitrile/10% water) was added. ② The mixture was sonicated for 5 minutes and allowed to stand at −40°C for 1 hour. ③ Under low-temperature conditions, the supernatant was centrifuged at 12,000 g for 15 minutes and transferred to a small bottle for testing. ④ The target compounds were separated by chromatography using the Vanquish ultra-high-performance liquid chromatography system. ThermoFisher Q Exactive HF-X mass spectrometer was used for mass spectrometry data acquisition. ⑤ The original data were converted using ProteoWizard software and annotated with the mass spectrometry database after processing. ⑥ Differential metabolites were visualized in the form of volcano plots and radar charts. By analyzing the enriched pathways and topology of differential metabolites, the pathways with the highest correlation to metabolite differences were identified and displayed in a rectangular tree diagram. The above results were explained in terms of the AOP-treated group compared to the AOP-control group.

### 16S rRNA sequencing analysis

① Collected oral and fecal samples from each group of mice under sterile conditions and stored them at −80°C for later use. ② Extracted total DNA according to the instructions of the TIANamp Bacteria DNA kit, and measured the purity and concentration of DNA using a NanoDrop spectrophotometer. Stored the extracted DNA at −80°C for later use. ③ Guangdong Magigene Biotechnology Co., Ltd. utilizes the PE250 strategy on the Illumina NovaSeq 6000 platform, employing primers 515F (GTGCCAGCMGCCGCGGTAA) and A806R (GGACTACVSGGGTATCTAAT) for high-throughput sequencing of the V4–V5 region of the bacterial 16S rRNA gene. ④ Sequence processing was performed after library construction. The sequencing results were annotated and clustered using the UPARSE algorithm (17). ⑤ α-diversity analysis, β-diversity analysis, and differential analysis of some species composition were based on the OTU table.

### Metagenomic sequencing analysis

① The sequencing was performed by Guangdong Magigene Biotechnology Co., Ltd. Used the ALFA-SEQ DNA Library Prep Kit to generate sequencing libraries and added index codes. Evaluated the library quality using the Qubit 4.0 Fluorometer (Life Technologies, Grand Island, NY) and the QSEP400 High-Throughput Nucleic Acid Protein Analysis System (Houze Biology Technology Co., Houze Biology Technology, China). After quality control of the library, the library was sequenced on the Illumina Novaseq 6000 platform, producing paired-end reads with a length of 150 base pairs. ② Species annotation was performed by comparing the sequencing data to the NCBI-NR database, and functional annotation was performed by comparing the sequencing data to the KEGG database.

### Co-inertia analysis

To help explain the relationship between the microbiome and metabolomics data sets, we integrated them using a multivariate method called co-inertia analysis (CIA) (18). Principal component analysis (PCA) was applied to each individual data set as input to CIA. The sorting of the two data sets with the highest covariation was determined to identify shared biological trends between the two data sets. CIA was performed using the ade4 package in R (4.2.1).

### Pearson correlation analysis

In order to investigate whether there is a correlation between the microbiota in the gut and oral cavity, this study conducted correlation analysis using the major microbial genera in the gut, oral cavity, AOP-control, and AOP-treated groups. A *P* value of less than 0.05 was considered statistically significant, and Pearson correlation analysis was performed using the Hmisc package in R (version 4.2.1).

### Statistical analysis

The data are presented as mean ± SD of independent samples. Analysis of variance and Mann–Whitney *U* tests were used for analyzing parametric or non-parametric data, respectively. Student's *t*-test was used for comparing between two groups. *P* < 0.05 was considered statistically significant. The criteria for metabolomics data analysis used a cutoff of *P* < 0.05 for Student's *t*-test and a variable projection importance greater than one for the first principal component of the orthogonal projections to latent structures discriminant analysis (OPLS-DA) model. Correlation analysis was performed by calculating the Spearman correlation coefficient, and a heat map was drawn for variables with |*r*| > 0.8 and *P* < 0.05.

## RESULTS

### Inhibition of γδ T affects gut metabolism in periodontitis promoting OSCC

In our prior research (9), we observed that oral microbiota from periodontitis can facilitate OSCC development by activating γδ T. In the AOP-treated group, which consisted of OSCC mice treated with periodontitis and UC7-13D5 injection to inhibit γδ T, the tumor weight was significantly decreased in comparison to their respective controls without γδ T inhibition. Furthermore, the AOP-treated group exhibited a lower γδ T cell percentage in tumor tissues and lower IL-17 expression in serum in comparison to their respective controls. These OSCC mouse parameters are presented in Table S1.

**Fig 1:**
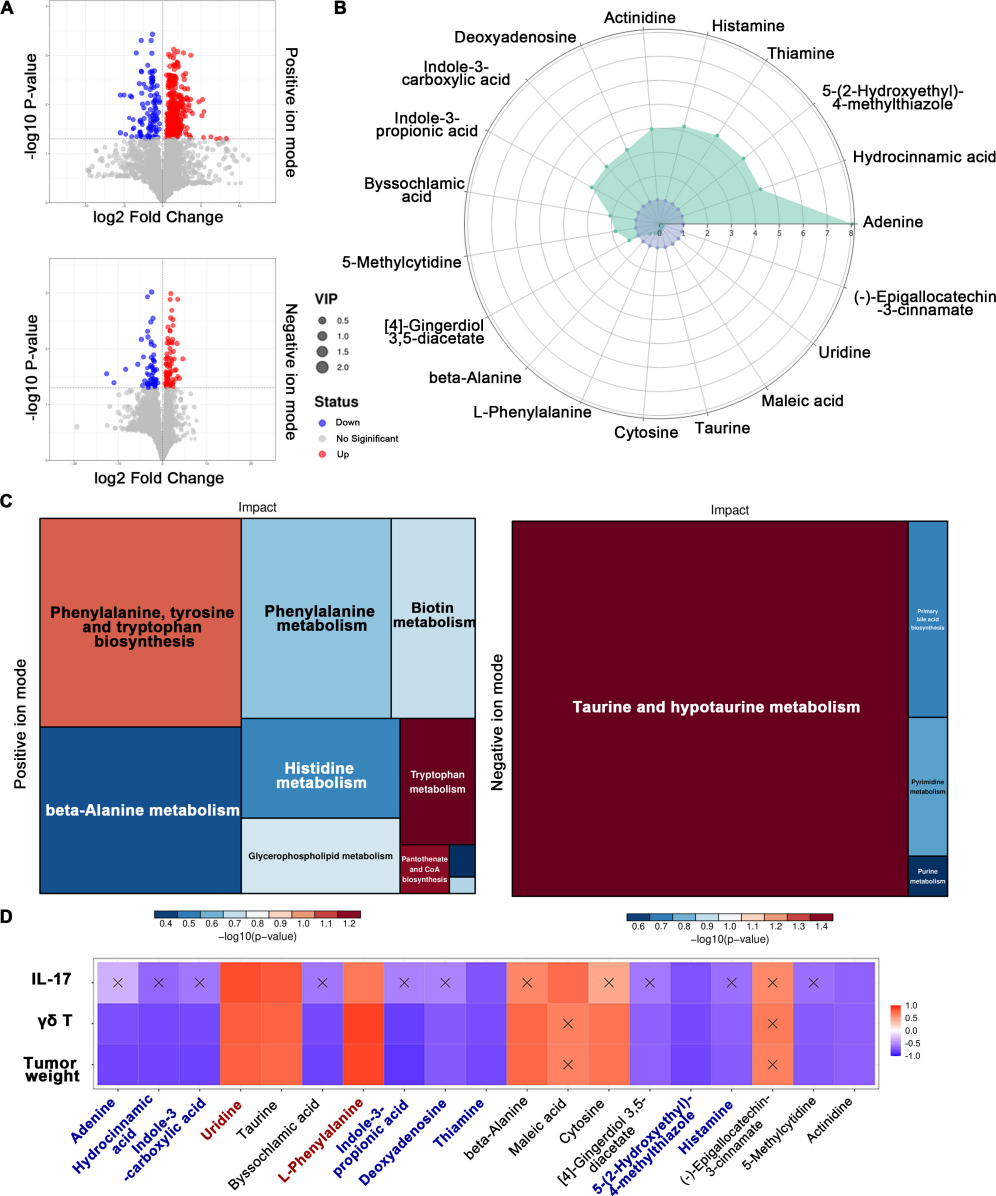
The effects of γδ T cell inhibition on the fecal metabolome of mice. (**A**) The volcano plot displays differential metabolites between the AOP-treated and AOP-control groups. The *x*-axis represents fold changes in each substance, and the *y*-axis represents the *P* value of the student *t*-test. The size of the scatter plot represents the VIP value of the OPLS-DA model. Red dots represent upregulated metabolites, and blue dots represent downregulated metabolites. (**B**) The radar plot shows trends in changes in the levels of differential metabolites. The ratios of the main differential metabolites were calculated, and a radar plot was made after logarithmic transformation with a base of 2. The purple shadow represents the AOP-control group, and the green shadow represents the AOP-treated group. (**C**) KEGG pathway enrichment analysis indicates 14 affected metabolic pathways. The size of the square represents the impact factor. The left panel represents the positive ion mode, and the right panel represents the negative ion mode. (**D**) Correlations between fecal metabolites, IL-17, γδ T cells, and tumor weight. The correlation coefficient (*r* value) is represented using a square color map, with red indicating positive correlation and blue indicating negative correlation. The darker the color, the higher the correlation. An "X" in a square indicates that the correlation analysis is not statistically significant.

To further identify key biomarkers for γδ T-immunotherapy in periodontitis-associated OSCC, this study conducted non-targeted liquid chromatography-mass spectrometry metabolomics analysis on the feces of mice in the AOP-control and AOP-treated groups, which identified 838 and 300 metabolites in positive and negative ion modes, respectively. After comparing the metabolic compositions of the AOP-treated and AOP-control groups, the differentially expressed metabolites were presented in a volcano plot (Fig. 1A), revealing significant differences in 530 metabolites (positive ion mode) and 147 metabolites (negative ion mode). Both positive and negative ion modes were used in this experiment for metabolite analysis. A radar chart was employed to display a set of metabolites with the most significant changes in content (Fig. 1B). Among them, the AOP-treated group exhibited a significant increase in the content of adenine, hydrocinnamic acid, 5-(2-hydroxyethyl)−4-methylthiazole, thiamine, histamine, actinidine, deoxyadenosine, indole-3-carboxylicacid, indole-3-propionicacid, byssochlamicacid, 5-methylcytidine, and [4]-gingerdiol3,5-diacetate, when compared to the AOP-control group. Meanwhile, the content of beta-alanine, L-phenylalanine, cytosine, taurine, maleicacid, uridine, and (-)-epigallocatechin3-cinnamate decreased significantly. The fecal metabolites adenine (19), hydrocinnamic acid (20), indole-3-carboxylic acid (21), deoxyadenosine (22), thiamine (23), and histamine (24), which are enriched in the AOP-treated mice, have been shown to have inhibitory effects on cell proliferation. Additionally, 5-(2-hydroxyethyl)−4-methylthiazole (25) and indole-3-propionic acid (26) are associated with anti-inflammatory effects. Conversely, the observed reduction of uridine (27, 28) and L-phenylalanine (29) in the feces of AOP-treated mice is associated with an increased risk of cancer.

To gain a better understanding of the metabolic pathways involved in the promotion of OSCC through the oral microbiome in periodontitis by γδT cells, significant changes in metabolites were analyzed using KEGG pathway enrichment analysis (Fig. 1C). The results revealed that the inhibition of γδT cells in mice with periodontitis-associated OSCC affected a total of 14 metabolic pathways, primarily involving taurine and hypotaurine metabolism, pyrimidine metabolism, primary bile acid biosynthesis, purine metabolism, phenylalanine, tyrosine, and tryptophan biosynthesis, beta-alanine metabolism, phenylalanine metabolism, and biotin metabolism, among others. Of these pathways, cell signaling and environmental stress response were identified as the most relevant biological processes.

This experiment conducted correlation analysis between the identified differential metabolites, the expression of IL-17, the abundance of γδ T, and tumor weight. The results were visualized using a heatmap (Fig. 1D). The metabolites adenine, hydrocinnamic acid, indole-3-carboxylic acid, deoxyadenosine, thiamine, histamine, 5-(2-hydroxyethyl)−4-methylthiazole, and indole-3-propionic acid, which have been reported to have inhibitory effects on cell proliferation or inflammation, were found to be significantly and negatively correlated with the abundance of γδ T. Conversely, uridine and L-phenylalanine, which have been associated with a high risk of cancer, showed significant and positive correlation with the abundance of γδ T. After obtaining the matching information of differentially expressed metabolites in each group, we performed pathway search and regulatory interaction network analysis on the KEGG database of the corresponding species. The results of the regulatory analysis are presented as a network plot (Fig. S2).

In the context of OSCC with periodontitis, targeting γδ T resulted in a substantial elevation in the levels of metabolites associated with anti-cancer effects and a concomitant decrease in those promoting cancer in the gut. This observation highlights the potential involvement of host metabolism in the immunotherapeutic efficacy of γδ T cell-targeting strategies and underscores the impact on multiple tumor-related metabolites. These findings further suggested that these metabolites might serve as crucial biological markers for the precise and effective immunotherapy of OSCC in the presence of periodontitis.

### Targeting γδ T improves gut microbiota homeostasis in periodontitis promoting OSCC

**Fig 2:**
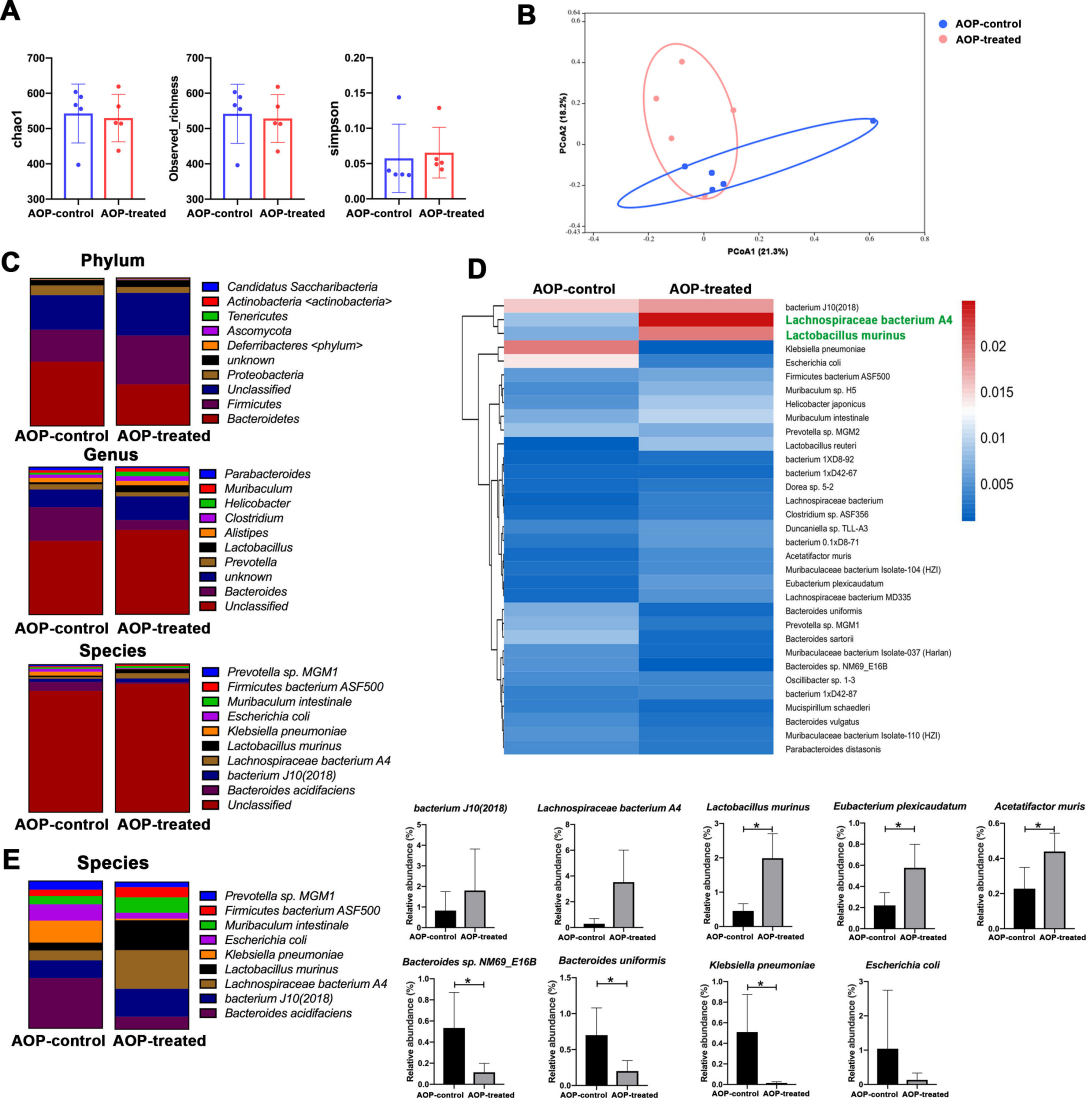
Analysis of diversity and abundance of gut microbiota in mice. (**A**) α-diversity of gut microbiota in each group of mice, as measured by Chao index, Observed richness, and Simpson index. (**B**) β-diversity of gut microbiota in each group of mice shown as a PCoA plot of UniFrac distances. (**C**) Abundance of major phylum, genus, and species of gut microbiota in mice. (**D**) Heatmap showing the relative abundance of 33 major classified bacterial species within each group. (**E**) Analysis of inter-group differences in major gut microbiota at the species level. *P < 0.05.

In this study, fecal samples from both the AOP-control and AOP-treated groups of mice were subjected to 16S rRNA sequencing, resulting in a data set of 734,467 high-quality sequences and identifying 5,352 OTUs. Although analysis using the Chao1, Observed_richness, and Simpson indices revealed no significant differences in α-diversity of gut microbiota between the two groups (Fig. 2A), the PCoA analysis showed that the targeted γδ T cell immunotherapy had a notable impact on the composition of gut microbiota, with significant differences in β-diversity of gut microbiota observed between the AOP-control and AOP-treated group mice (Fig. 2B).

Furthermore, metagenomic sequencing was conducted to gain further insight into the gut microbiota of the two groups of mice, revealing that the dominant phyla were *Bacteroidetes* (35.4%), *Firmicutes* (27.2%), and *Proteobacteria* (5.2%). At the genus level, *Bacteroides* (12.3%), *Prevotella* (2.6%), *Lactobacillus* (2.5%), *Alistipes* (2.5%), and *Clostridium* (2.3%) were the most abundant genera. Among the species, *Bacteroides acidifaciens* (2.6%), *bacterium J10(2018)* (1.7%), *Lachnospiraceae bacterium A4* (1.7%), *Lactobacillus murinus* (1.3%), *Klebsiella pneumoniae* (1.0%), *Escherichia coli* (0.9%), *Muribaculum intestinale* (0.86%), *Firmicutes bacterium ASF500* (0.6%), and *Prevotella* sp. MGM1 (0.55%) were the most prevalent (Fig. 2C).

Based on the genus-level sequencing results of intestinal microorganisms, the experiment conducted a correlation analysis between all genus-level classifications and the IL-17 protein concentration, γδ T cell abundance, and tumor weight (Fig. S3). The analysis indicated a significant positive correlation between *Bacteroides* and the abundance of γδ T, while *Lactobacillus* was significantly negatively correlated with the abundance of γδ T. During the targeted inhibition of γδ T in the treatment of OSCC with periodontitis, the abundance of the beneficial intestinal bacteria (30) *Lactobacillus* increased, which was significantly correlated with the IL-17 protein concentration and γδ T cell abundance.

The study also presented a heatmap that displays the relative abundance of 33 major bacterial species identified in the AOP-control and AOP-treated group. The top three major bacterial species in the gut of mice in the AOP-control group were *bacterium J10(2018)*, *E. coli*, and *K. pneumoniae*, while *bacterium J10(2018)*, *L. bacterium A4*, and *L. murinus* were enriched in the gut of mice in the AOP-treated group (Fig. 2D). Statistical analysis of the gut microbiota at the species level revealed significant differences between the two groups, with the mice receiving targeted γδ T cell immunotherapy exhibiting a significant enrichment of *L. murinus*, *Eubacterium plexicaudatum*, and *Acetatifactor muris* in the gut, along with a significant decrease in *K. pneumoniae*, *Bacteroides uniformis*, and *Bacteroides* sp. NM69_E16B (Fig. 2E).

Overall, the results suggested that in the promotion of OSCC progression by periodontitis oral microbiota, targeting the inhibition of γδ T could simultaneously improve gut homeostasis and enrich probiotics.

### The impact of inhibiting γδ T on microbiota functional genes in promoting OSCC by periodontitis oral microbiota

**Fig 3:**
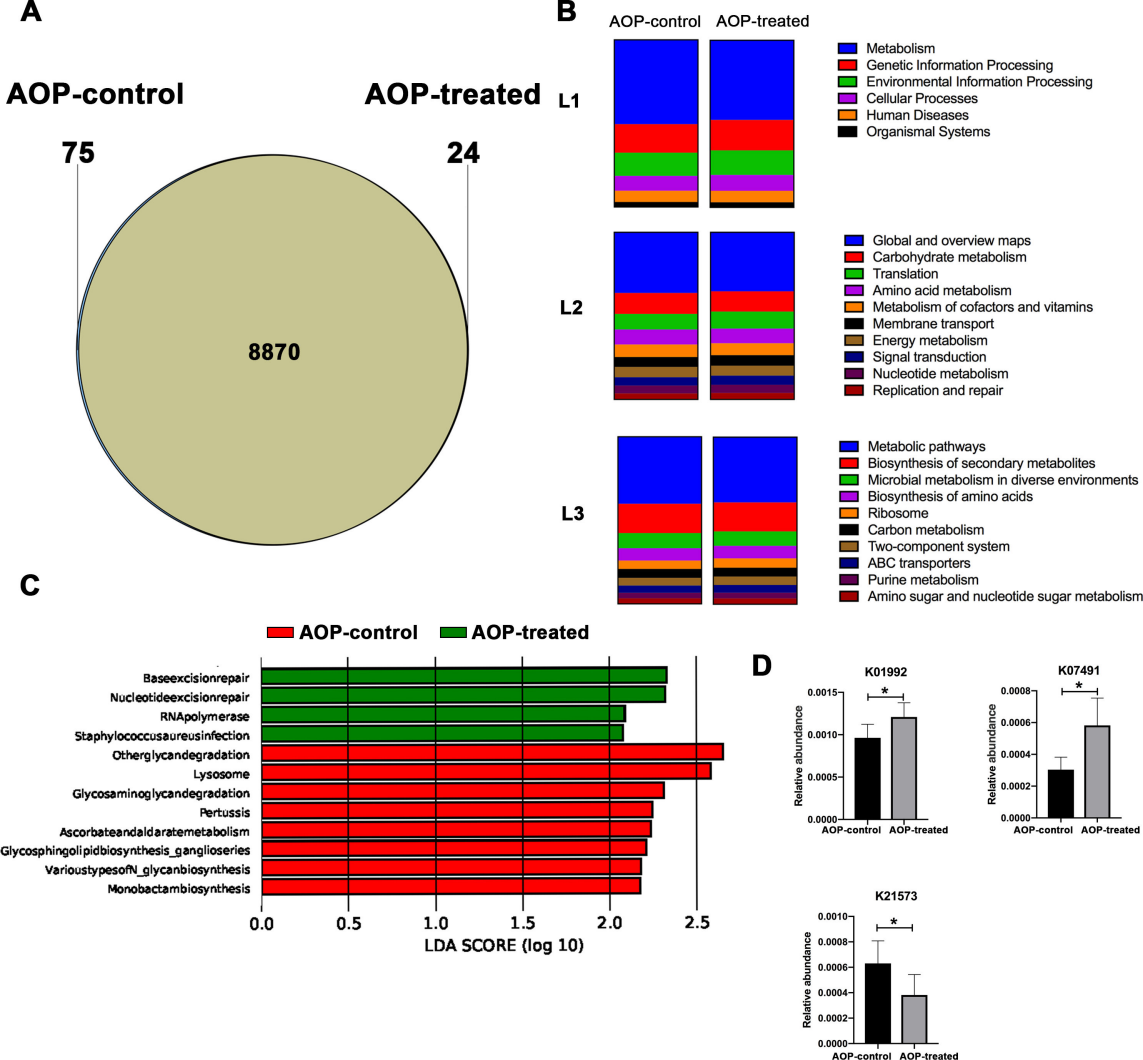
Pathway enrichment analysis and abundance ratio. (**A**) The Venn diagram intuitively shows the number of common genes among groups. (**B**) The pathway distribution of AOP-control group and AOP-treated group at different classification levels. (**C**) The differences between groups in terms of pathways. The graph shows the LDA values of the different species, with colors representing the corresponding groups. The length of the bars in the graph represents the contribution of each species to the differences (LDA Score). LDA score is greater than 2. (**D**) Analysis of inter-group differences at the KO level. *P < 0.05.

In the context of periodontitis oral microbiota promoting OSCC, inhibiting γδ T resulted in microbiota functional gene profiles that were almost identical between the two groups, with some differences still present, as shown by the Venn diagram (Fig. 3A). To investigate the specific impact of inhibiting γδ T on functional genes, KEGG pathway enrichment analysis was performed, which indicated that the pathway distribution of the AOP-control and AOP-treated groups was similar, with metabolic pathways being the largest proportion at the L1 level. Visualization of pathway abundance at the L2 level revealed that both groups had similar abundance levels, with amino acid metabolism, translation, and carbohydrate metabolism exhibiting higher abundance levels (Fig. 3B).

The analysis of the pathways that differed significantly between the AOP-control and AOP-treated groups were performed. The results showed that the AOP-treated group had significantly higher base excision repair, nucleotide excision repair, RNA polymerase, and *Staphylococcus aureus* infection pathways, while the AOP-control group had significantly higher other glycan degradation, lysosome, glycosaminoglycan degradation, pertussis, ascorbate and aldarate metabolism, glycosphingolipid biosynthesis-ganglio series, various types of N-glycan biosynthesis, and monobactam biosynthesis pathways (Fig. 3C). Moreover, this study conducted further analysis of the pathways and key proteins (enzymes) that showed significant differences between the AOP-control and AOP-treated groups, revealing differences in the relative abundance of proteins K01992, K07491 (a protein that plays an important role in genome evolution, adaptive evolution, and the immune system) (31, 32), and K21573 between the two groups (Fig. 3D).

Additionally, this study investigated the correlation between these microbiota functional genes and the concentration of IL-17, the abundance of γδ T, and tumor weight. There were significant negative correlations between the abundance of γδ T and K07491 (Fig. S4). Moreover, K07491 and K07483 (a transposase that can cause changes in genome structure and gene regulation) (33) were significantly negative correlated with the concentration of IL-17. Furthermore, significant positive correlations were observed between γδ T and the abundance of K21573 (involved in the degradation and utilization of starch) (34, 35), K07165 (a type of receptor protein in bacteria that can sense signals in the environment), and K07485 (a type of transposase that can catalyze transposition reactions).

The results indicated that the inhibition of γδ T cells could impact the functional gene profiles of oral microbiota associated with periodontitis. Furthermore, the observed differences in functional gene profiles and their relationship with the immune system highlight the potential importance of these factors as therapeutic targets for the prevention and treatment of OSCC.

### Correlation between gut metabolites and microbiota in periodontitis oral microbiota-promoted OSCC mice with γδ T suppression

**Fig 4:**
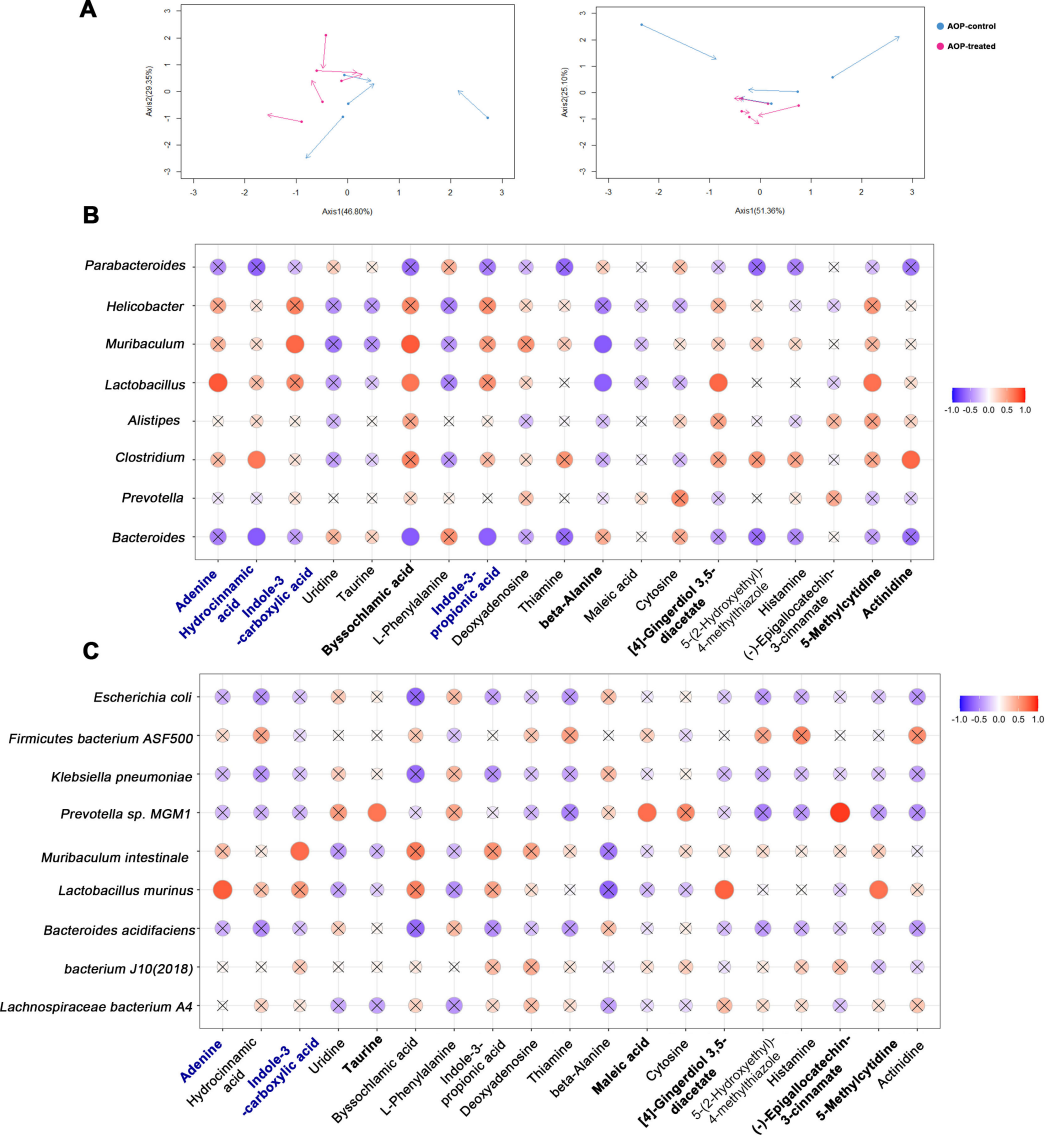
Association between gut metabolites and microbes in mice. (**A**) The correlation between major microbiota at the genus (left) or species (right) level and major changed metabolites in the intestine is presented, where the starting points represent metagenomic features and the endpoints represent metabolomic features. The blue dots represent the AOP-control group, while the red dots represent the AOP-treated group. (**B–C**) The correlation between major microbiota at the genus (**B**) or species (**C**) level and major changed metabolites in the intestine is shown. The horizontal axis displays the major microbiota, while the vertical axis shows the different metabolites. The strength of the correlation is represented by the color of the circle, with red indicating a positive correlation and blue indicating a negative correlation. The darker the color, the stronger the correlation. An "X" inside the circle indicates that the correlation analysis is not statistically significant.

To investigate the potential correlation between the impact of γδ T suppression on alterations in microbiota and metabolites in the mouse intestine, this study performed bioinformatics analyses on the metabolomic and metagenomic data. PCA was conducted on both data sets, retaining two characteristic values, to visualize the correlation between the two. CIA was then carried out to explore the differences in metabolites and microbiota between the control and experimental groups. This analysis revealed a certain correlation between changes in intestinal metabolism and microbiota at the genus and species levels in both the AOP-control and the AOP-treated mice (Fig. 4A).

The study further investigated the relationship between the main microbiota at the genus or species level and the major metabolites that underwent changes (Fig. 4B and C). A comprehensive analysis revealed that *L. murinus* of the *Lactobacillus* genus, a type of intestinal microbiota, is associated with changes in multiple metabolites, as well as with the concentration of IL-17 protein and the abundance of γδ T. The key metabolite associated with *L. murinus* is adenine, which has been reported to have potential anticancer effects and is also correlated with the abundance of γδ T (36).

By conducting bioinformatics analysis, we found that there is a certain correlation between changes in intestinal metabolism and microbiota. Moreover, we identified *L. murinus* as the key microbiota associated with changes in multiple metabolites, the concentration of IL-17 protein, and the abundance of γδ T. Adenine, a key metabolite associated with *L. murinus*, was also found to have potential as a therapeutic target for preventing and treating OSCC. These findings suggest that targeting *L. murinus* and adenine may offer promising approaches for the treatment of OSCC.

### Investigating the impact of inhibiting γδ T on resistance genes, virulence factors, and microbiota interaction

**Fig 5:**
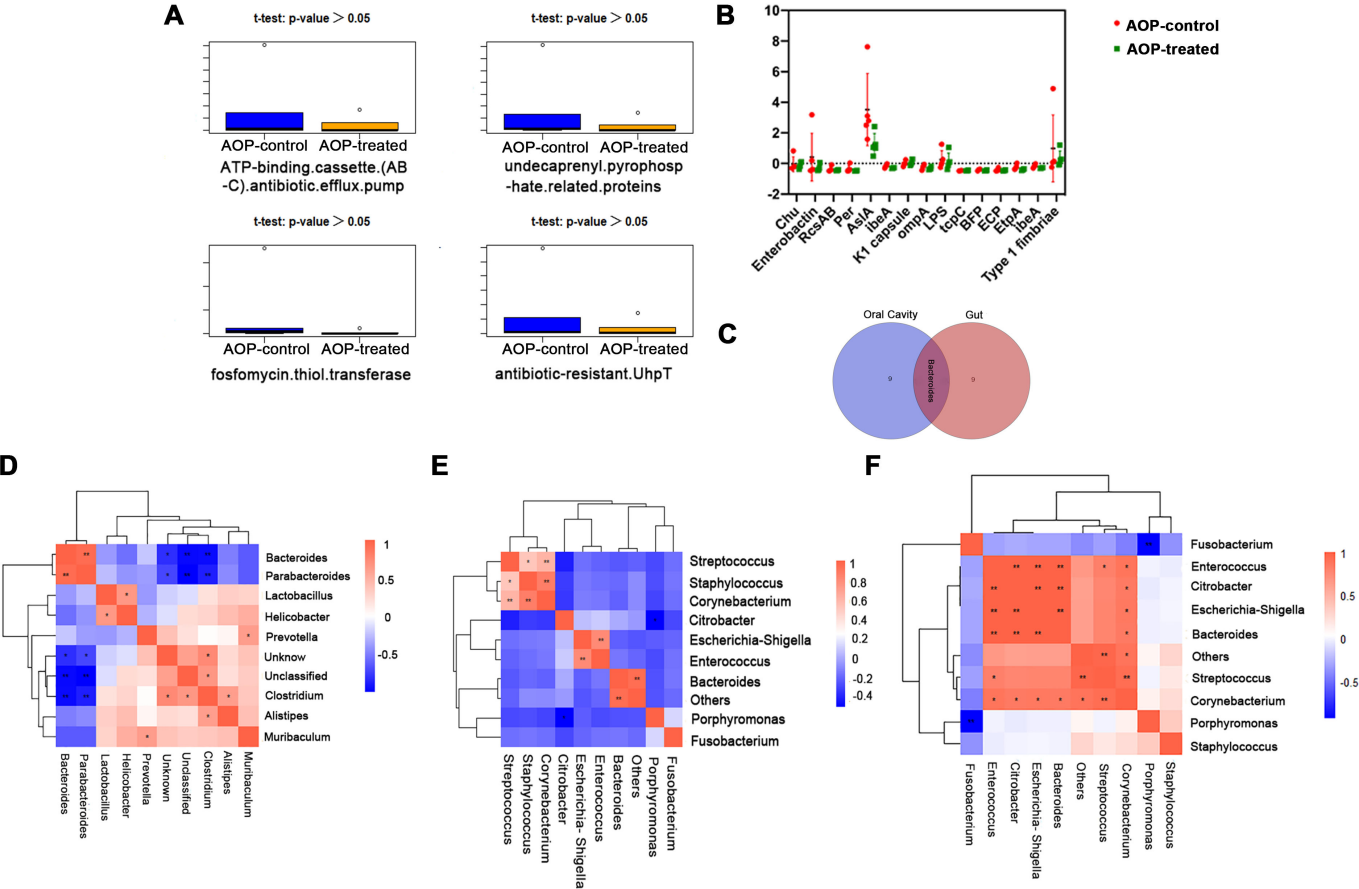
Differences in *t*-tests for mouse resistance genes and virulence factors, as well as at the microbial genus level. (**A**) *t*-Test of resistance genes between AOP-control group (blue) and AOP-treated group (yellow). (**B**) *t*-Test of independent bacterial factors. (**C**) The Venn diagram shows the number of major microbial genera at the level of the mouse gut and oral cavity. (**D–F**) Correlation analysis of microbial genera at the level of the gut (**D**) or oral cavity (**E**) or oral bacteria in the AOP-control group versus the AOP-treated group (**F**).

In this study, we conducted a thorough investigation of the impact of inhibiting γδ T on resistance genes in periodontitis-associated OSCC. *t*-Tests were performed on the top four highest abundance resistance genes in the AOP-control and AOP-treated groups, and no significant difference in resistance genes was observed between the two groups (Fig. 5A). However, further *t*-test analysis on virulence factors revealed a significant difference in *AslA*, an acidic lipopeptide found in some pathogens (Fig. 5B). AslA can degrade important molecules of the host immune system, and help the pathogen evade immune surveillance. Moreover, AslA can also cleave important proteins on the surface of host cells, such as platelet-agglutinating factor, interfering with normal host cell function (37, 38).

In addition, we investigated the oral and gut microbiota and found significant differences at the genus level, with Bacteroides being the only overlapping genus (Fig. 5C). We also examined the correlation at the genus level between the oral and gut microbiota. In the gut (Fig. 5D), *Bacteroides* was positively correlated with *Parabacteroides* and negatively correlated with *Clostridium*. In the oral cavity (Fig. 5E), the three pairs of *Streptococcus* and *Corynebacterium*, *Staphylococcus* and *Corynebacterium*, and *Escherichia-Shigella* and *Enterococcus* are positively correlated, while *Porphyromonas* and *Citrobacter* are negatively correlated. Furthermore, analysis of the correlation at the genus level of microbiota in the oral cavity of mice with periodontitis-associated OSCC (Fig. 5F) showed that *Bacteroides* was significantly positively correlated with *Enterococcus*, *Citrobacter*, and *Escherichia-Shigella*, *Enterococcus* was positively correlated with *Citrobacter*, *Escherichia-Shigella*, and *Bacteroides*, and *Streptococcus* was positively correlated with *Corynebacterium*.

Our study highlights the significance of inhibiting γδ T in reducing virulence factors in periodontitis-associated OSCC. Furthermore, our analysis revealed significant differences in the microbiota in the oral cavity and intestine. These findings provide valuable insights into the complex interactions among microbiota and virulence factors, and might contribute to the development of effective treatment strategies for OSCC.

## DISCUSSION

γδ T cells have strong plasticity and can polarize from one phenotype to another. It has been reported that γδ T cells can polarize into γδ T17 cells (producing only IL-17), γδ T1/17 cells (producing both IFN-gamma and IL-17), γδ T1 cells (producing both IFN-gamma and TNF-alpha), and γδ T2 cells (producing IL-4). γδ T cells and their secretion of IL-17 play an important role in cancer cell growth (39), while γδ T cells can also weaken the anti-tumor ability of other immune cells (40, 41). Due to the plasticity of γδ T cells, they can be regarded as “friend” or “foe” in different cancer contexts.

In certain cancers, γδ T cells can shape an immune-suppressive tumor microenvironment through their own accumulation and function, or by enhancing the activity of other immune-suppressive cells, thereby promoting cancer progression. Our previous research has also demonstrated that γδ T cells are a key upstream node in the pro-carcinogenic effect of periodontal disease-associated oral microbiota.

Previous studies have shown that there is a correlation between the gut microbiota and γδ T cells. The gut microbiota can inhibit IL-17 production in cecal γδ T cells, and metabolites derived from these microbiota can reduce IL-17 production by gut γδ T cells (13). In this study, there was a significant positive correlation between the abundance of γδ T cells and K07491, and a significant negative correlation with K21573. K07491 is the REP-associated tyrosine transposase (rayT), which belongs to the HUH superfamily of nucleases, whose members promote replication or mobilization of various mobile genetic elements (viruses, plasmids, and insertion sequences) (42). In addition to the conserved residues involved in DNA cleavage catalysis, RAYT also carries characteristic structural motifs absent from typical IS200/IS605 transposases. In one-third of the cases, the DNA sequence flanking the rayT gene is arranged within bacterial interspersed mosaic elements (BIMEs) (32), and recent studies have shown that DNA gyrase and DNA polymerase I can specifically recognize BIME DNA *in vitro*. These results suggest that BIMEs may play a role in the functional organization of bacterial nuclei (43). K21573 is a TonB-dependent starch-binding outer membrane protein SusC, which is essential for bacterial growth on starch (35). The process of nutrient acquisition by bacteria is executed by protein machines on the cell membrane. For many bacteria in the gut microbiota, this process is accomplished by a protein complex consisting of a substrate-binding protein (SusD) and a channel transporter protein (SusC) (34). Therefore, we believe that targeting γδ T cells as a therapeutic approach can induce changes in the metabolic microenvironment within the intestines.

It is worth noting that γδ T cells can respond to cytokine stimulation and change their function. Artificially altering the cytokine balance in the tumor immune microenvironment may be another new immunotherapy method targeting γδ T cells. Researchers advocate routine testing of IL-17 levels before starting γδ T cell adoptive immunotherapy to predict the immune status. In summary, whether using γδ T cell expansion or targeting γδ T cells, attention should be paid to their function and the regulation of cytokine balance in the tumor microenvironment.

Researchers generally agree that there is a mutual interaction between the microbiome and γδ T cells, which can affect tissue homeostasis and disease development. The role of the microbiome in the development and function of γδ T cells has been a focus of research. However, the reverse regulation of the microbiome by γδ T cells has been underestimated by researchers. Although there are reports that lung γδ T cells in cancer patients are closely positively correlated with lung microbiome alpha diversity (44), the impact of γδ T cells on the microbiome, especially in the oral microbiome, has not been fully determined. Studies have shown that ablating γδ T cells significantly alters the diversity of the oral microbiome, and similar microbiome changes have been reported in IL17R-deficient mice (45), supporting the importance of IL-17 signaling in γδ T cell homeostasis. However, the bidirectional relationship between γδ T cells and the microbiome, especially the effect of γδ T cells on the microbiome, has not been fully determined. This study first analyzed the impact of ablating γδ T cells on gut ecology in promoting OSCC progression by the oral microbiome in periodontitis. The sequencing data of mouse feces supported that γδ T cells would reshape the host microbiome ecology in a reverse manner. After inhibiting γδ T cells, the content of anticancer metabolites in the intestines of tumor-bearing mice increased, the content of cancer-promoting metabolites decreased, and the probiotics were enriched in the intestines. Furthermore, it was found that *L. murinus*, adenine, and γδ T cell abundance were closely related.

In summary, the influence of host microbiota should be considered in the process of targeting γδ T cell therapy for OSCC with concurrent periodontitis. First, the impact of environmental changes on the reshaping of γδ T cell function should be carefully considered, including the influence of oral microbiota structure on γδ T cell numbers and function, which in turn affects tumor development. In addition, the reverse effect of γδ T cells on the intestinal ecosystem is also worth exploring and utilizing.

## Data Availability

All data generated or analyzed during this study are included in this published article. The raw sequence data (PRJCA017220) of metagenomic and 16S rRNA reported in this paper have been deposited in the Genome Sequence Archive (Genomics, Proteomics & Bioinformatics 2021) in National Genomics Data Center (Nucleic Acids Res 2022), China National Center for Bioinformation / Beijing Institute of Genomics, Chinese Academy of Sciences (GSA: CRA011605) (GSA: CRA011276) that are publicly accessible at https://ngdc.cncb.ac.cn/gsa. The metabolomics data reported in this paper have been deposited in the OMIX, China National Center for Bioinformation / Beijing Institute of Genomics, Chinese Academy of Sciences (https://ngdc.cncb.ac.cn/omix: accession no. OMIX004320) (46, 47).

## AFTER EPUB

[This article was published on 23 January 2024 with errors in the Funding table in the PDF. The funding information was corrected in the current version, posted on 31 January 2024.]

## SUPPLEMENTAL MATERIAL

Supplemental material msystems.00777-23-s0001.docx Supplemental figures and table.

ASM does not own the copyrights to Supplemental Material that may be linked to, or accessed through, an article. The authors have granted ASM a non-exclusive, world-wide license to publish the Supplemental Material files. Please contact the corresponding author directly for reuse.
