# Supplementary material for: Metabolomics and metagenomics reveal the impact of γδ T inhibition on gut microbiota and metabolism in periodontitis-promoting OSCC
Source: mSystems. 2024 Jan 23;9(2):e00777-23. doi: 10.1128/msystems.00777-23 (PMC10878065; doi:10.1128/msystems.00777-23)
Supplement: Supplemental material — Supplemental figures and table. [file msystems.00777-23-s0001.docx]

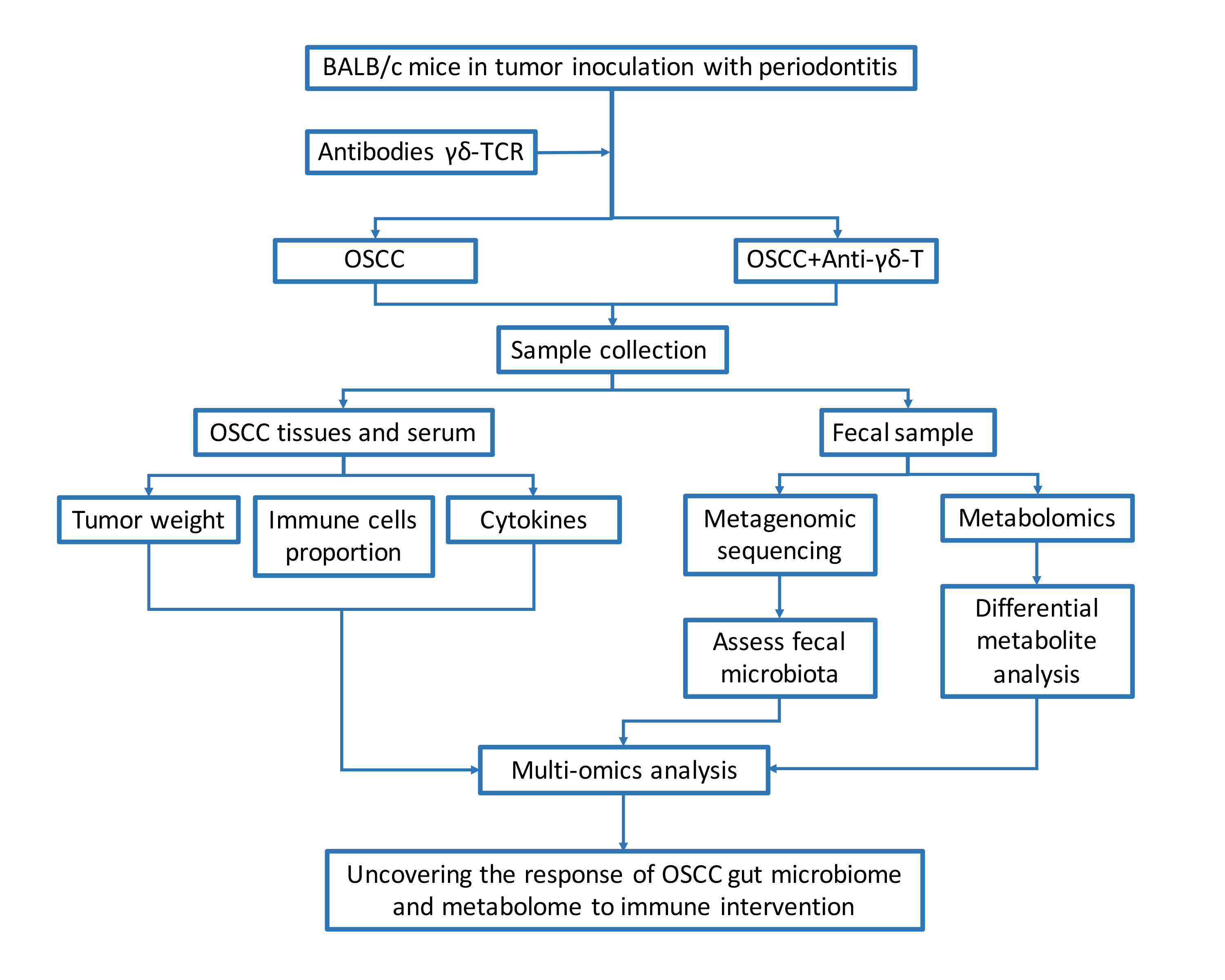


**Supplemental Figure 1 Experimental flowchart**

**
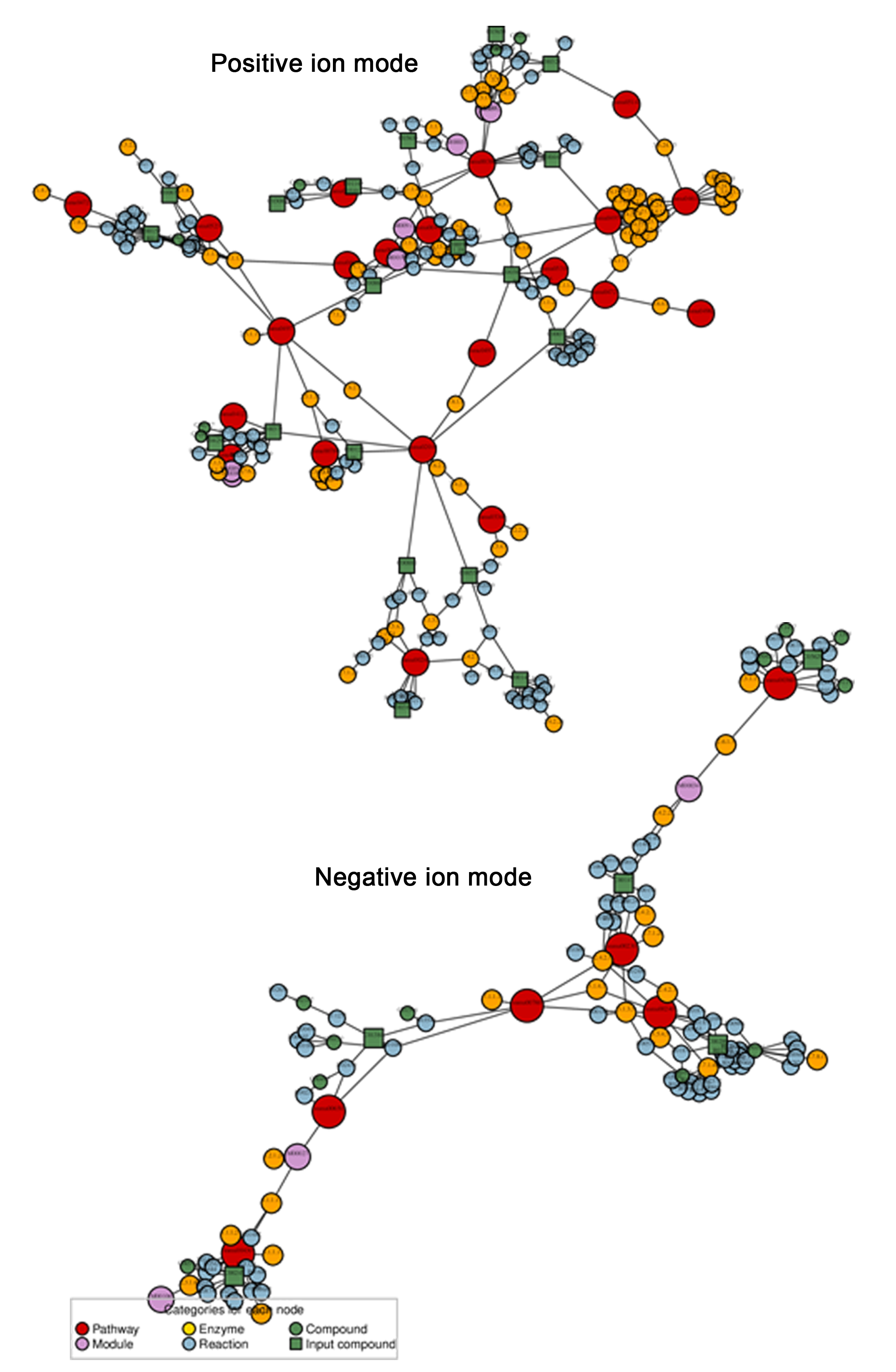
**

**Supplementary figure 2: Regulatory interaction network.** In the figure, red dots represent a metabolic pathway, yellow dots represent enzyme information related to a substance regulation, green dots represent a background substance of a metabolic pathway, purple dots represent a type of substance molecular module information, blue dots represent a chemical interaction reaction of a substance, and green squares represent differential substances obtained from this comparison.


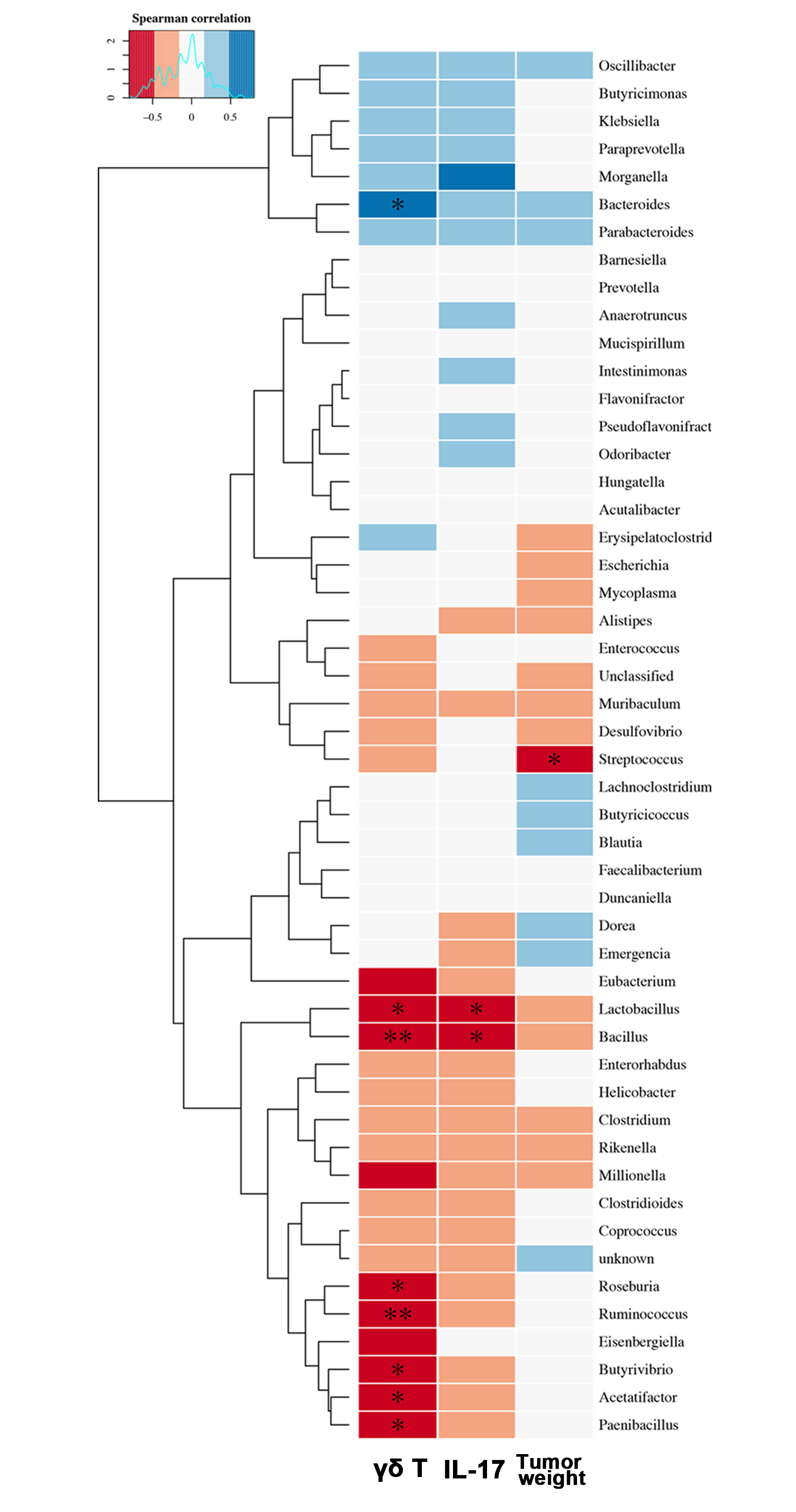


**Supplemental Figure 3 Correlation between mouse gut microbiota at the genus level and IL-17, γδ T cell abundance, and tumor weight.** The correlation coefficient (r) is represented using a square color chart, with red indicating negative correlation and blue indicating positive correlation. The darker the color, the higher the correlation. An asterisk (*) in the square indicates statistical significance, *: *p* < 0.05 and **: *p* < 0.01.


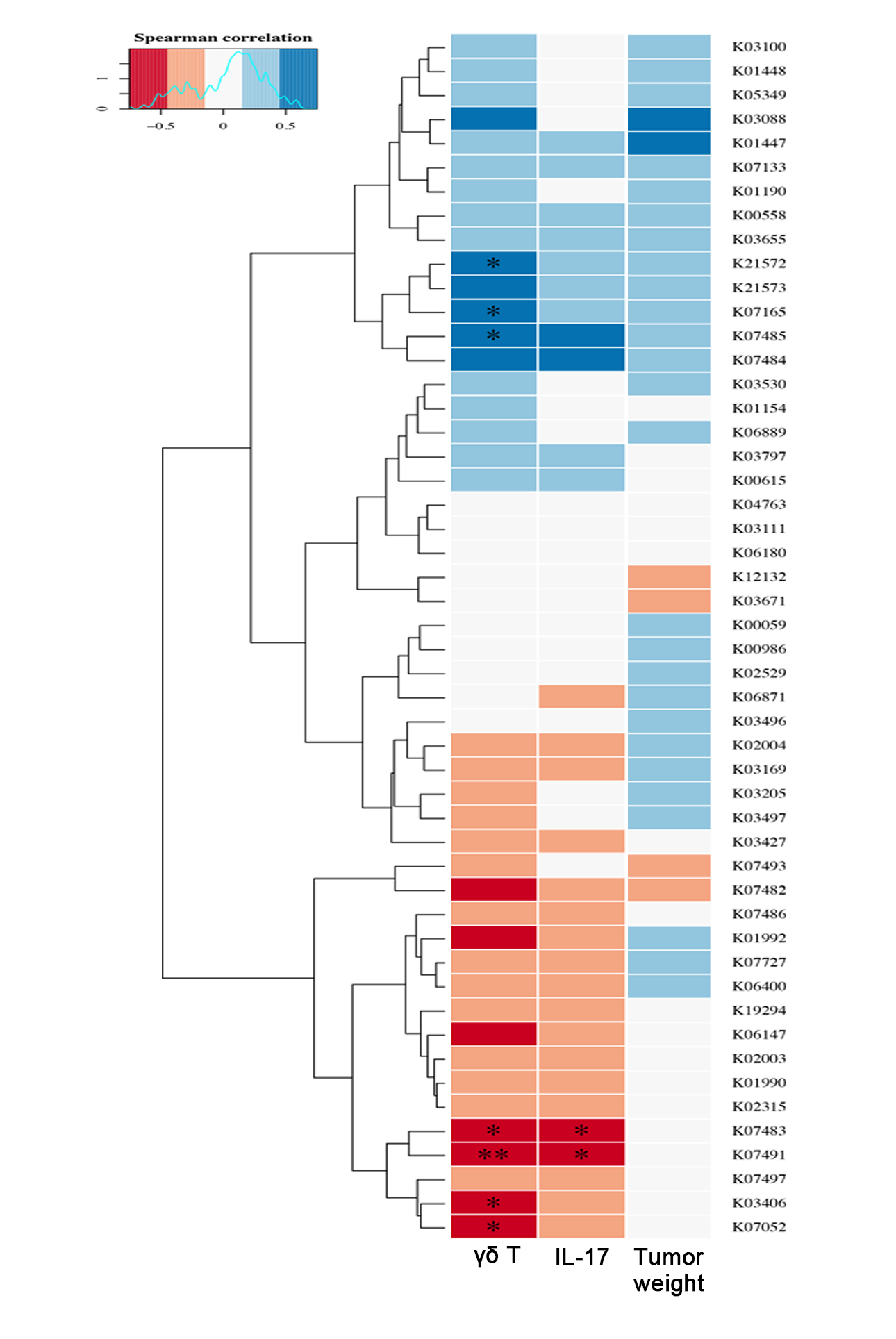


**Supplemental Figure 4 Correlation between differentially pathways and IL-17, γδ T cells, and tumor weight.** The correlation coefficient (r) is represented using a square color chart, with red indicating negative correlation and blue indicating positive correlation. The darker the color, the higher the correlation. The K series number represents a gene, and each number represents a homologous gene across all species. An asterisk (*) in the square indicates statistical significance, *: *p* < 0.05 and **: *p* < 0.01.

**Supplemental Table 1 Summary characteristics**

| Parameter | Group | mean ± SEM | n | Significance  (p value) | t | df |
| --- | --- | --- | --- | --- | --- | --- |
| Tumor weight (g) | AOP-control | 0.7617 ± 0.06332 | 6 | p = 0.0149 | 3.000 | 9 |
|  | AOP-treated | 0.2980 ± 0.1526 | 5 |  |  |  |
| γδ T (% T cells) | AOP-control | 41.68 ± 0.9410 | 5 | p < 0.0001 | 18.77 | 8 |
|  | AOP-treated | 18.06 ± 0.8352 | 5 |  |  |  |
| IL-17 (pg/ml) | AOP-control | 128.4 ± 4.689 | 5 | p = 0.0004 | 5.746 | 8 |
|  | AOP-treated | 73.01 ± 8.416 | 5 |  |  |  |

Data were analyzed by Student's t test.
